# Brain Death and Organ Donation Rates in a City Hospital: A Retrospective Study

**DOI:** 10.7759/cureus.4006

**Published:** 2019-02-04

**Authors:** Sibel Yılmaz Ferhatoglu, Nihan Yapici

**Affiliations:** 1 Anesthesiology, Siyami Ersek Cardiothoracic Surgery Hospital, Istanbul, TUR

**Keywords:** brain death, determining brain death, transplantation, ancillary test, icu

## Abstract

Introduction

Although organ donation rates have been increasing over the years, the lack of organ donation remains the most important problem in transplantation. By changing strategies, the Cekirge City Hospital in Bursa/Osmangazi has achieved a cadaveric donor rate of 24.9 per one million individuals in 2016; this rate is 21.5 in England, 20.9 in Norway, 14.7 in the Netherlands, and 10.6 in Germany.

Methods

Brain death cases were retrospectively evaluated between January 1, 2011, and December 31, 2016.

Results

There were a total of 137 brain death cases. Three of eight cases, five of 12 cases, three of 13 cases, 13 of 25 cases, 16 of 29 cases, and 21 of 50 cases became a donor in 2011, 2012, 2013, 2014, 2015, and 2016, respectively.

Conclusion

Deceased organ donation rates have increased over the years; however, the number of brain dead patients and the acceptance of organ donation by families have been increasing, but the percentage of brain death donations did not increase. We suggest that the reason for this situation is that well-trained and educated physicians diagnose more brain death cases and have a greater desire to treat end-stage organ failure patients, but the tendency of the public to donate has not increased as hoped. Donation and transplantation rates may be increased with a combination of well-trained, educated, and dedicated physicians with public education.

## Introduction

Brain functions were first described by Mollaret et al. in 1959 using a concept similar to the modern definition of brain death. Mollaret and Goulon differentiated "coma dépassé" from other "coma states," such as a vegetative state [1]. Brain death determination criteria were first published in 1968, a year after the first heart transplantation. Attitudes toward brain death differ greatly between countries worldwide, and there is no global consensus in diagnostic criteria [2-3]. The American Academy of Neurology published guidelines and practice parameters to help physicians determine brain death. The guidelines of the American Academy of Neurology were published in 1995 and updated in 2010 [4-5]. The brain death determination guidelines of the Turkish Neurological Society were published in 2014 [6]; they are in accordance with the law on the Regulation on Organ and Tissue Transplantation of the Turkish Ministry of Health in 2012 and with the guidelines of the American Academy of Neurology in 2010.

Recognizing and determining brain death is important because the number of patients waiting for organ transplantation increases day by day, and the deceased organ donation rates remain insufficient throughout the world. Hesitancy in brain death diagnosis by physicians and the ethical concerns of the families of candidate donor patients are the two largest obstacles in finding the right organ for the receiving patient whose survival depends on the transplantation.

Cardiac death during evaluation, donor instability, and death during organ recovery are medical problems that also result in the loss of donor candidate patients. Therefore, teaching and encouraging anesthesiologists, neurologists, and neurosurgeons on how to perform a rapid diagnosis of brain death are crucial.

Organ transplantation is the only option for most patients affected by end-stage organ failure, and health policymakers have paid increasing attention to this subject during the last decade. Medical care for end-stage organ failure patients presents a large financial burden on the overall budget of most countries. Over the last decade, the definitive treatment of these patients through organ transplantation has decreased health expenditures; therefore, increasing cadaver donation rates has become more important for health politicians. The universal shortage in cadaver donation has increased living donor rates, particularly for liver and kidney transplantation. A total of 30,973 transplants from 15,064 donors were performed in the United States of America in 2015, and more than 121000 thousand patients affected by end-stage organ failure were waiting for a transplant in 2015 [7]. As the situation is evaluated by the donor candidate patients and their families, age and ethical concerns create an obstacle for organ transplantation. Moreover, less than 1% of all people who die in hospitals can be considered for organ donation because they must die under specific circumstances [8].

The aim of this study was to investigate the strategies for determining brain death retrospectively and the statistics of brain death and organ donation in Cekirge State Hospital, Bursa, Turkey.

## Materials and methods

The brain death determining strategy at our hospital

In our hospital, we have developed a brain death determining strategy that works in harmony with organ transplantation coordinators, neurologists, and neurosurgeons. The organ transplantation coordination committee consists of two members. One of them has to be a physician and the other one may be a physician or a nurse. We, the anesthesiologists, and the organ transplantation coordinators examine all intensive care unit patients every day, particularly patients in tertiary intensive care units (Glasgow Coma Scale ≤ 7). Patients with head trauma, cerebrovascular disease, and post-cardiopulmonary resuscitation are also followed up closely. When brain death is suspected, first the anesthesiologists and then the neurologists perform a neurologic examination. If the patient is suitable for testing, the anesthesiologists perform the apnea test. If the apnea test is aborted, an ancillary test is performed. The ancillary tests that we can use in our hospital are computed tomography (CT) angiography and magnetic resonance imaging (MRI) angiography. Magnetic resonance imaging angiography is the preferable ancillary test. Even when the apnea test is positive, we perform an ancillary test because it can reduce the time of observation. Finally, a committee consisting of two doctors, one anesthesiologist, and one neurologist or one neurosurgeon confirms the brain death and, then, brain death can be declared to the patient’s family.

All procedures are performed according to the Helsinki declaration.

Statistical analysis

Number Cruncher Statistical System (NCSS; NCSS, LLC., Utah, US) 2007 was used for the statistical analysis. The mean, standard deviation, and percentage values were calculated as descriptive statistics. Qualitative data were compared using Pearson’s chi-square test. A linear-by-linear association test was used to examine the changes over the years. Statistical significance was accepted when p < 0.05.

The author of this study, Sibel Yilmaz Ferhatoglu was appointed to Siyami Ersek Cardiothoracic Surgery Hospital from Cekirge State Hospital during the writing process of this article. All patients included in the study were followed-up and treated in the Cekirge State Hospital, Bursa, Turkey, and the assignment of the first author to another hospital did not change the methodology of the study.

## Results

Patient demographics

A total of 137 brain death cases were assessed in this study, including 82 male patients (59.9%) and 55 female patients (40.1%). The mean age was 54.23 ± 17.15 years. The mean age of the male patients was 51.04 ± 16.67 years, and the mean age of the female patients was 59.0 ± 16.89 years. All of the patients were over 18 years old. According to blood types, A Rh-positive was the most common blood group (35.8%). None of the brain death cases had an AB Rh-negative blood type. In both genders, intracerebral hemorrhage (28.47%) was the most common cause of brain death. In females, cerebrovascular ischemia (15.33%) was the second most common cause of brain death, and in males, traumatic subarachnoid hemorrhage (16.06%) was the second most common cause of brain death (Table 1).

**Table 1:** Cause of brain death, age, and sex of the cases

	Number of cases	Percentage of cases	Male (N)	Female (N)	Age (mean ± standard deviation)
Acute subdural hematoma	10	7.30	10	0	47.30 ± 14.97
Hypoxic encephalopathy	17	12.41	9	8	57.53 ± 18.98
Intracranial tumor	1	0.73	0	1	75
Intracerebral hemorrhage	39	28.47	23	16	57.18 ± 16.31
Cerebrovascular ischemia	21	15.33	9	12	61.67 ± 9.94
Cerebral contusion	6	4.38	6	0	34.50 ± 21.70
Spontaneous subarachnoid hemorrhage	18	13.14	8	10	56.22 ± 15.15
Traumatic subarachnoid hemorrhage	22	16.06	16	6	45.82 ± 18.18
Cerebrospinal fluid fistula/pneumocephalus	1	0.73	0	1	44
Epidural hematoma	1	0.73	1	0	42
Subacute subdural hematoma	1	0.73	0	1	66

Apnea test

The apnea test was completed and positive in 123 cases (89.9%); in 14 cases (10.1%), the apnea test was aborted primarily because of arrhythmia.

Ancillary test

In determining brain death, the preference for the use of an ancillary test increased from 2011 to 2016. In 2011, there was no ancillary test confirmation because there was no ancillary test in our hospital. After 2011, ıf there was no contraindication transportation of the patient for an ancillary test, the ancillary test has done. In 2016, 42 cases of brain death were confirmed with ancillary tests (84%). Thirty-one of the 137 cases were determined as brain death only clinically (22.6%), whereas 106 were determined clinically and confirmed with magnetic resonance imaging angiography (77.4%).

Brain death diagnosis time

The time from the hospitalization of the patient to the diagnosis of brain death is four days. The mean brain death diagnosis time was 4.40 ± 4.05 days (range 0–26 days). The time is from the hospitalization of the patient to the diagnosis of brain death. Intracranial tumor and cerebrovascular ischemia were the most quickly diagnosed causes.

Donation acceptance and rejection

**Figure 1:**
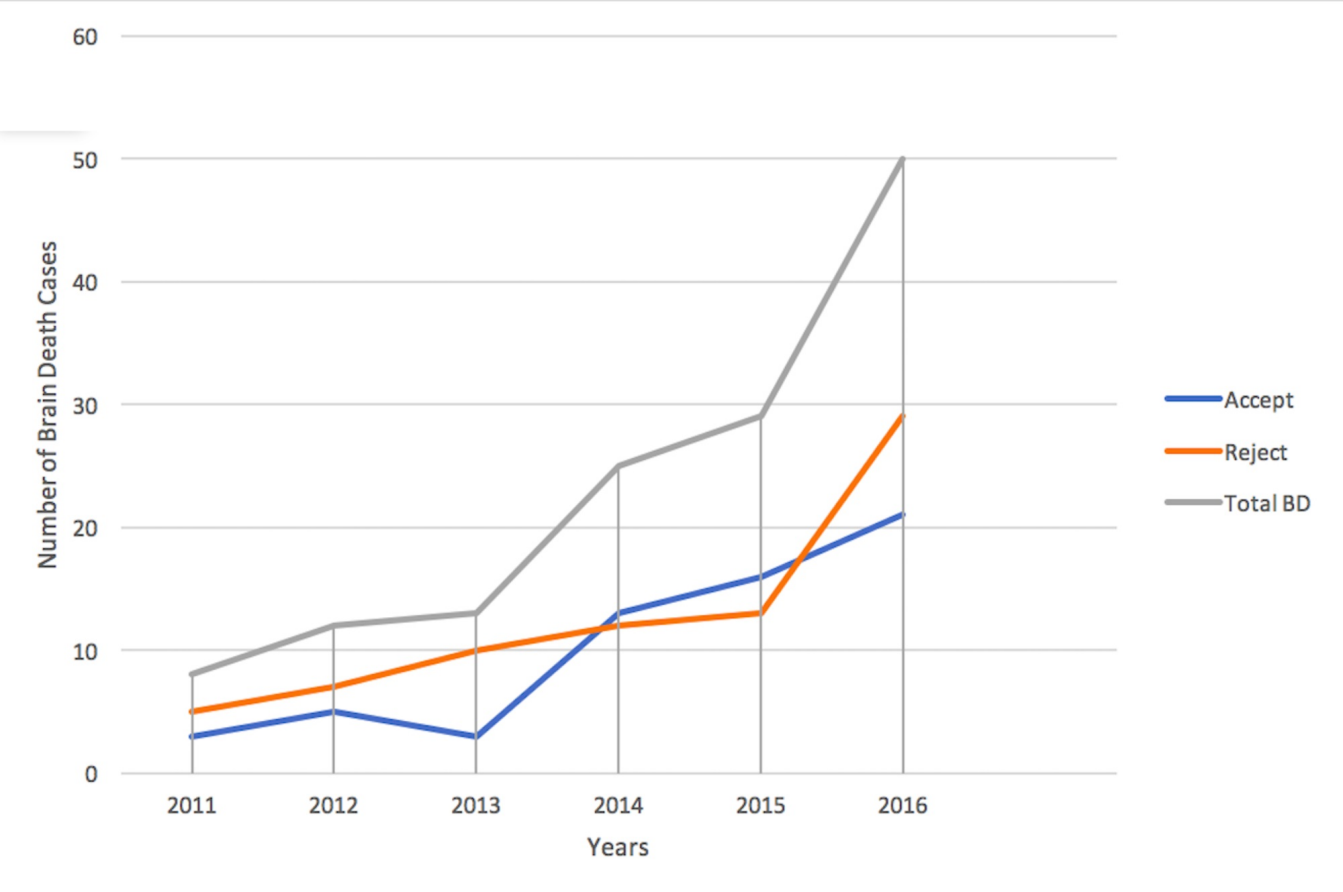
Total number of brain deaths, acceptance, and rejection for each year

Of the 137 patients diagnosed with brain death, organs were donated in 61 patients, 26 (42.6%) of whom were female and 35 (57.4%) of whom were male. The acceptance and rejection rates for each year are shown in Figure 1.

Harvested organs

The liver and kidney were the most frequently donated organs, followed by the heart. Over the six-year study period, 61 livers, 100 kidneys, 11 hearts, five corneas, two lungs, and one pancreas were harvested.

Brain death determination rate in our hospital

The brain death determination rate in our hospital in 2011 was 0.4%, which increased to 2% in 2016. The brain death determination rate in 2016 was statistically significantly higher than the rates in 2012, 2013, and 2014, with p = 0.001, p < 0.001, and p = 0.007, respectively (Tables 2-3).

**Table 2:** The number of patients staying in the intensive care unit and the number of brain death cases per year

	2011	2012	2013	2014	2015	2016
Intensive care unit stay	1976	1594	2062	2381	2060	2500
Number of brain death cases	8	12	13	25	29	50

**Table 3:** Comparison of organ donation rates by year intervals Pearson’s chi-square test *p < 0.05 **p < 0.01 OR: odds ratio

Years	p	OR (95% CI)
2011-2012	0.173	1.866 (0.761, 4.576)
2011-2013	0.323	1.561 (0.646, 3.774)
2011-2014	0.019*	2.610 (1.175, 5.800)
2011-2015	0.002**	3.513 (1.602, 7.702)
2011-2016	<0.001**	5.020 (2.375, 10.614)
2012-2013	0.656	0.836 (0.381, 1.838)
2012-2014	0.339	1.399 (0.701, 2.793)
2012-2015	0.062	1.882 (0.958, 3.701)
2012-2016	0.001**	2.690 (1.428, 5.068)
2013-2014	0.130	1.672 (0.853, 3.278)
2013-2015	0.013*	2.251 (1.167, 4.342)
2013-2016	<0.001**	3.217 (1.743, 5.938)
2014-2015	0.278	1.346 (0.786, 2.305)
2014-2016	0.007**	1.923 (1.186, 3.119)
2015-2016	0.127	1.429 (0.901, 2.267)

Deceased organ donation rate per million people

Deceased organ donation rate increased significantly after 2014. The donor rate in 2016 was 24.9 per million people (Table 4).

**Table 4:** Organ donation rate / per million people *: Population of Bursa, Osmangazi district

	Donation	Population * Per million people value**
2011	3	789.575 3.7
2012	5	792.219 6.3
2013	3	802.620 3.7
2014	13	813.262 15.9
2015	16	826.742 19.3
2016	21	841.756 24.9

## Discussion

Organ transplantation is the only solution for most patients affected by end-stage organ failure. Trying to cure these patients by medical treatment is difficult or impossible in most cases and is expensive. An economic analysis of transplantation shows that transplantation is both cheaper and more successful than medical treatment. “Increasing organ transplantation rates by 50% could achieve a further cost saving of two hundred million euros per annum,” as stated in the United Kingdom organ transplantation strategy 2020 review. Increasing organ donation by simple precautions is the main aim of all efforts. The U.K. Organ Donation Taskforce increased rates in after-death organ donation by 50% between 2007/2008 and 2012/2013 by highlighting three key areas: donor identification and reveal, donor coordination, and organ retrieval arrangements [9].

There are different national organ transplantation programs. Spain has developed a successful organ donation program called the Spanish Model of Donation and Transplantation. In this model, organ transplantation coordinators play an important role: they are responsible for enhancing organ donation in their hospital and representing the hospital level of the organizational network [10]. Other successful organ donation models have been established in the United States of America and Australia [10-11]. In our hospital, teamwork between organ donation coordinators, anesthesiologists, neurologists, and neurosurgeons and the development in determining a brain death strategy over time were the key areas contributing to our success. We achieved a cadaveric donor rate of 24.9 per one million individuals in 2016; this rate was 21.5 in England, 20.9 in Norway, 14.7 in the Netherlands, and 10.6 in Germany [12]. We believe that developing special hospital strategies may increase donation rates.

In our hospital, 44.5% of brain death cases became donors between 2011 and 2016. Most of the patients diagnosed with brain death were male, similar to other studies [3,13-16]. Trauma is more common in males in some studies in our country, but there is no information about gender in the Turkish Statistical Institute data. The reason for the increased incidence of a brain death diagnosis in males is that most accidents are accompanied by heavy head trauma [17-18].

Intracerebral hemorrhage was the most common cause of brain death in our study, similar to the studies by Escudero et al. [13] and Pandey et al. [3]. However, many studies reported different primary causes of brain death [14-15,19]. The difference in the most common cause of brain death in various studies may be a result of using different classifications for the cause of brain death.

The brain death determination method differs in each country throughout the world [2-3,13] In our hospital, the clinical determination rate was only 22.6%, whereas the clinical determination and ancillary test confirmation rate was 77.4%. Since there was no contraindication of transportation, we chose to perform an ancillary test to determine brain death in many cases to reduce the observation time. Pandey A. et. al [3] pointed to the variability in diagnosing brain death between 2011 and 2015 in their hospital. In their study, an apnea test was completed in 49.5% of patients, and an ancillary test was completed in 29.8% of patients. Escudero et al. discussed the different practices in brain death diagnoses with a multicenter study that had a clinical determination rate of only 5% and an ancillary test rate of 95% with at least one test [13].

In our study, the family acceptance of donation after brain death was 44.5%. In three other studies done in our country, the family acceptance rates for being a donor were 34.2%, 29%, and 8.7%, respectively [14-15,19]. Ethnic and religious aspects are the most important factors affecting the organ donation rate [11,16,20].

In our hospital, deceased organ donation rates have increased over time: in 2011, 37.5% of brain death cases became donors, whereas in 2016, 42% of the cases became donors. Although the number of brain death cases have been increasing, the percentage of organ donation acceptance of families has not been increasing as expected. We hypothesize that this occurs because well-trained and educated physicians experience more brain death cases and have a greater desire to treat end-stage organ failure patients, but the tendency for the public to donate has not increased as hoped. We suggest that both physician awareness and public awareness are important topics for organ donation.

According to the Turkish Ministry of Health, there are 24,588 patients currently waiting for organ donation who are on the transplant list in 2016: 21,474 patients for a kidney, 2164 for a liver, 762 for a heart, 272 for a pancreas, 11 for a kidney and a pancreas, three for heart valves, and two for intestines. In 2016, 1999 brain death cases were declared and 564 of these became donors, resulting in 4921 transplantations performed. In 2008, there were two hundred six transplantations; thus, the transplantation rate has increased by 23.8 times in eight years since then [21].

Since there are few studies in the literature that can be used for comparing the data of the present study, the retrospective nature and short follow-up period of the present study are the limitations. On the other hand, we believe that the study gives very important ideas about organ donation and expectations about education related to this topic.

## Conclusions

Recognizing and determining brain death are two important steps for organ donation. Creating teamwork and harmony between organ donation coordinators, anesthesiologists, neurologists, and neurosurgeons is necessary for the determination of brain death and increasing organ donation rates. Also, the education of health employees and the public is another important issue to achieve better donation rates. Governments and international organizations should work harder on this health problem. We think a national brain death strategy must be identified and governments must pay more attention to this subject.
